# BMP4 promotes hepatocellular carcinoma proliferation by autophagy activation through JNK1-mediated Bcl-2 phosphorylation

**DOI:** 10.1186/s13046-018-0828-x

**Published:** 2018-07-16

**Authors:** Ganlu Deng, Shan Zeng, Yanling Qu, Qingqing Luo, Cao Guo, Ling Yin, Ying Han, Yiyi Li, Changjing Cai, Yaojie Fu, Hong Shen

**Affiliations:** 1 Department of Oncology, Xiangya Hospital, Central South University, Changsha, 410008 Hunan China; 2 National Clinical Research Center for Geriatric Disorders, Xiangya Hospital, Central South University, Changsha, 410008 Hunan China; 3 Key Laboratory for Molecular Radiation Oncology of Hunan Province, Xiangya Hospital, Central South University, Changsha, 410008 Hunan China

**Keywords:** BMP4, Autophagy, Hepatocellular carcinoma, Proliferation, JNK1

## Abstract

**Background:**

Autophagy is a conserved catabolic process with complicated roles in tumor development. Bone morphogenetic protein 4 (BMP4), a member of the transforming growth factor (TGF-β) family of regulatory proteins, plays a crucial role in human malignancies. However, whether BMP4 contributes to the regulation of autophagy in hepatocellular carcinoma (HCC) progression remains elusive.

**Methods:**

Functional analysis of BMP4 on HCC proliferation and autophagy was performed both in vitro and in vivo in HepG2 and HCCLM3 cells. Autophagic activity was estimated by Western blot for autophagic marker proteins and by transmission electron microscopy (TEM). Transfection of mRFP-GFP-LC3 adenovirus was applied to observe autophagic flux and high content screening was used for quantification. The signaling pathway of BMP4-regulated HCC proliferation and autophagy was investigated by Western blot.

**Results:**

BMP4 treatment promoted HCC cells proliferation and induced autophagy. The in vivo xenograft model supported that BMP4 overexpression promoted the growth of HCC cells and autophagy induction while BMP4 knockdown exerted the opposite effect. 3-MA pre-treatment or knockdown of Beclin-1 (BECN1) blocked HCC autophagy by decreasing the expression of LC3-II and subsequently attenuated BMP4-induced autophagy and cells proliferation enhanced by BMP4 in vitro and in vivo. Mechanistic study revealed that the induction of autophagy by BMP4 was mediated through activating the JNK1/Bcl2 pathway. Furthermore, the JNK1 inhibitor and knockdown of JNK1 could attenuate autophagy induced by BMP4 and eliminated BMP4-promoted HCC cells growth.

**Conclusions:**

BMP4 promoted HCC proliferation by autophagy activation through JNK1/Bcl-2 signaling.

**Electronic supplementary material:**

The online version of this article (10.1186/s13046-018-0828-x) contains supplementary material, which is available to authorized users.

## Background

Hepatocellular carcinoma (HCC) is the second cause of cancer-related death worldwide with increasing incidence [1]. Therapeutic strategies for HCC, including surgical resection, ablation, systemic or infusional chemotherapy, have improved the survival of HCC patients. However, aggressive malignancy and late diagnosis result in a poor prognosis for HCC patients, with a five-year survival rate of less than 14% [2]. A great effort has been made to study the carcinogenesis and pathogenesis of HCC, however further research into new treatment approaches and the underlying molecular mechanism are needed.

Autophagy is an evolutionarily conserved physiological process whereby unnecessary intracellular components, such as misfolded proteins and dysfunctional organelles, are sequestered in double-membrane vesicles (autophagosomes) and then transported to lysosomes for degradation and recycling. This process replenishes cells with energy to maintain cellular homeostasis [3]. Autophagy is ubiquitous in eukaryotic cells and plays a critical role in diverse pathophysiological conditions such as infection and tumor development [4]. The role of autophagy in carcinogenesis is variable [5]. Autophagy may suppress tumorigenesis in flatworm germline tumors by degrading toxic oncogenic proteins [6]. Research on mouse kidney iBMK cells and human bladder urothelial carcinoma suggest that autophagy promotes tumor proliferation and protects tumor cells from stresses like chemotherapy or hypoxia by maintaining cancer metabolism [7, 8]. The exact role of autophagy in chemosensitivity, cell death, and proliferation of HCC cells remains unclear.

Bone morphogenesis proteins (BMPs), a large subgroup of the transforming growth factor β (TGF-β) superfamily members, have been found to regulate multiple aspects of embryonic development, cell proliferation, differentiation, apoptosis, chemotaxis, and cell fate during embryonic development [9]. BMP ligands elicit their biologic effects by binding to the heteromeric complex of BMP receptors BMPR1 and BMPR2. Upon ligand binding, the heteromeric complex activates phosphorylation-dependent SMAD1/5/8 proteins, which can assemble into complexes with SMAD4 to translocate into the cell nucleus and modulate the transcription of target genes [10].

BMP signaling has been linked to pathogenesis of clinical disorders such as the activation of hepatic stellate cells (HSCs) and hepatic cirrhosis [11]. In recent years, BMPs have been implicated in multiple human malignancies. Emerging bodies of evidences indicate that the dysregulation of BMP4 has promoting effects on tumor proliferation, metastasis and drug-resistance [12–15]. Contrary evidences exist for the role of BMP4 in HCC progression. BMP4 knockdown had no significant impact on HCC proliferation [16]. Moreover, BMP4 additions have shown inhibitory effects on HCC progression [17]. In our previous study, we have shown that BMP4 enhanced the proliferation of HCC cells Bel-7402 and HCCLM3 by promoting G1/S cell cycle via ID2/CDKN1B signaling [14]. However, the exact effect of BMP4 on autophagy-regulated HCC progression needs to be explicit.

We carried out this study to determine the effects of BMP4 on autophagy-regulated HCC proliferation in vitro and in vivo, as well as the involved molecular signaling mechanisms.

## Methods

### Reagents and antibodies

Recombinant human BMP4 (120-05) or Noggin (120-10C) were purchased from PeproTech (Rocky Hill, NJ). 3-Methyladenine (3-MA) (S2767) and JNK inhibitor SP600125 (S1460) were purchased from Selleckchem (Houston, TX, USA). Rapamycin (Rapa, V900930) was purchased from Sigma Chemical Co. (St. Louis, MO, USA). Reagents were reconstituted and stored according to the manufacturer’s description. Antibodies against BMP4 (ab124715), LC3 (#4108), SQSTM1/p62 (ab56416), Beclin1 (BECN1, ab207612), JNK1 (ab213521), phospho-JNK (Thr183/Tyr185) (#4668), Bcl-2 (#4223), phospho-Bcl2 (Ser70) (#2827), LC3B (ab51520), Ki-67 (ab15580) and GAPDH (ab181602) were purchased from Cell Signaling Technology (Beverly, MA, USA) and Abcam (Cambridge, UK).

### Cell culture

Human normal hepatic cell line L02 (3131C0001000200006) and six HCC cell lines (3111C0001CCC000-035, − 679, − 674, − 376, 3142C0001000000316, SCSP-528) were obtained from the Cell Bank of Typical Culture Preservation Committee of Chinese Academy of Science, Shanghai, China. The cells were cultured in high glucose Dulbecco’s modified Eagle medium (DMEM) supplemented with 10% fetal bovine serum (FBS) (Gibco, Grand Island, NY), 100 U/mL penicillin sodium and 100 μg/mL streptomycin (Biotechnology, Beijing, China) at 37 °C under an atmosphere of 95% air and 5% CO_2_.

### Quantitative real-time reverse transcription polymerase chain reaction (qRT-PCR)

Total RNA was extracted using Trizol Reagent (15596018, Invitrogen, Carlsbad, CA) and the cDNA (1 μg per sample) was synthetized using the PrimeScript™ Kit (RR037A, TaKaRa Bio Inc., Otsu, Japan) according to the manufacturer’s protocols. qRT-PCR was performed in triplicate by SYBR Green fluorescent-based assay (638320, TaKaRa Bio Inc.) on a ViiATM7 RT-PCR system (Applied Biosystems, Carlsbad, CA). The primers for real-time PCR were listed as follows: BMP4 Forward: 5’-CTCCAAGAATGGAGGCTGTAGGAA-3′; Reverse: 5’-CCTATGAGATGGAGCAGGCAAGA-3′; GAPDH Forward: 5’-CTGGGCTACACTGAGCACC-3′; Reverse: 5’-AAGTGGTCGTTGAGGGCAATG-3′; JNK1 Forward: 5’-TCTGGTATGATCCTTCTGAAGCA-3′; Reverse: 5’-TCCTCCAAGTCCATAACTTCCTT-3′. Relative mRNA expression levels were calculated by the 2^-ΔCt^ [ΔCt = Ct (targeting gene)-Ct (GAPDH)] method and were normalized to the internal control of GAPDH.

### Western blot

Total protein was extracted in RIPA lysis buffer (P0013B, Beyotime, Shanghai, China). 30 μg proteins per sample were separated by 12% SDS-PAGE and then transferred onto 0.2 μm PVDF membranes (Millipore, Bedford, MA) under 300 mA constant current for 1 h. The membranes were blocked in TBST (TBS with 0.5% Tween) containing 5% skim milk at 37 °C for 1 h and respectively incubated with the primary antibodies at 4 °C overnight followed by incubation with HRP-conjugated secondary antibody for 1 h at 37 °C. Detailed information for primary antibodies and secondary antibodies was provided in Additional file 1: Table S1. Signals were detected by the enhanced chemiluminescence kit Immobilon Western HRP substrate (WBKLS0500, Millipore), the bands were automatically visualized using a ChemiDoc XRS+ system (Bio-Rad, Hercules, CA) and quantitatively analyzed with Image Lab software (Bio-Rad). GAPDH protein expression was used as the internal control. Original blots were provided in the Additional file 1: Figure S1.

### Cell viability assay

Cells were pipetted into a 96-well plate (Costar, Cambridge, MA) at a density of 2.0 × 10^3^/well overnight and then were subjected to various indicated treatments. Cell viability was determined by Cell Counting Kit-8 (CCK-8, CK04-1000 T, Dojindo Molecular Technologies, Inc., Tokyo, Japan). Briefly, added 10 μL CCK-8 to each well and the absorbance at 450 nm was measured. Each assay was performed in triplicate.

### Colony formation assay

Cells were seeded in 60-mm dishes at a density of 1 × 10^3^/dish and then incubated with various treatments for 2 weeks to form colonies. Cells were fixed with methanol for 15 mins and stained with 0.1% crystal violet for 20 mins at room temperature. Colonies containing ≥50 cells were manually counted under a microscope. Each assay was performed in triplicate.

### Transmission electron microscopy (TEM)

The treated cells were fixed by 2.5% glutaraldehyde in 0.1 M phosphate buffer at 4 °C for 2 h and followed by 1% osmium tetroxide for 3 h. The samples were scraped and pelleted, dehydrated in a graded series of ethanol baths, infiltrated, and embedded in Epon resin. Ultrathin sections (70 nM) were cut by a Leica Ultracut Microtome then stained with uranyl acetate for 3 mins. The ultrastructures of cells undergoing autophagy were observed and captured under TEM (Hitachi HT-7700, Tokyo, Japan).

### Transient transfection of mRFP-GFP- LC3 adenovirus

To observe and analyze the autophagic flux, cells were transfected with a tandem fluorescent mRFP-GFP- tagged LC3 adenovirus according to the manufacturer’s instructions. Cells were seeded onto 96-well plates (clear bottom, black, Perkin-Elmer, Waltham, MA, USA) and allowed to approximately 50% confluence before transfection. HCC cells were incubated with 50 μL growth medium with mRFP-GFP-LC3 adenovirus (HB-AP2100001, HanBio Technology Co., Shanghai, China) at 30 multiplicities of infection (MOI). After 4 h, the adenovirus was moved off and the transfected cells were exposed to various indicated treatments.

### Imaging and analysis of autophagic flux

Cells transfected with mRFP-GFP-LC3 adenovirus after indicated treatments were fixed with 4% paraformaldehyde and washed three times with PBS in the dark. Image acquisition was performed using an Opera High Content Screening System (Perkin-Elmer). The data were analyzed using Columbus 2.3 software (Perkin-Elmer) performed by three channels (cellular nuclei defined by DAPI channel, cellular cytoplasm defined by GFP channel and RFP channel).

### BECN1 siRNA and transfections

Small interfering RNA (siRNA) targeting BECN1 was purchased from GenePhram (A10001, Shanghai, China). BECN1 was silenced using siRNA targeting the following sequences: sense 5′- GUGGAAUGGAAUGAGAUUATT-3′ and antisense 5′-UAAUCUCAUUCCAUUCCACTT-3′. The sequences of negative control were listed as follows: sense 5′-UUCUCCGAACGUGUCACGUTT-3′ and antisense 5′-ACGUGACACGUUCGGAGAATT-3′. Cells were seeded in 6-well plates overnight and then transfected with BECN1 siRNA or negative control siRNA separately using Lipofectamine® RNAiMAX Transfection Reagent (13778150, Invitrogen) according to the manufacturer’s instructions. After 48 h, the transfected cells were harvested for immunoblotting and subsequent assays.

### Knockdown of JNK1 by siRNA

siRNA targeting JNK1 was purchased from GenePhram (A10001, Shanghai, China). Sequences of siRNA targeting JNK1 or negative control were listed as follows: JNK1 sense 5′-GCCGACCAUUUCAGAAUCATT-3′ and antisense 5′-UGAUUCUGAAAUGGUCGGCTT-3′; negative control sense 5′-UUCUCCGAACGUGUCACGUTT-3′ and antisense 5′-ACGUGACACGUUCGGAGAATT-3′. Cells were seeded in 6-well plates overnight and then transfected with JNK1 siRNA or negative control siRNA separately using Lipofectamine® RNAiMAX Transfection Reagent (13778150, Invitrogen) according to the manufacturer’s instructions. After 48 h, the transfected cells were harvested for immunoblotting and subsequent cell viability assays.

### Lentivirus construction with overexpressed BMP4 and infection in HepG2 cells

The following oligo nucleotides (Forward: 5’-atatgaattcgccaccATGATTCCTGGTAACCGAATGCT-3′; Reverse: 5’-atatggatccTCAGCGGCACCCACATCCCTCTA-3′) were synthesized and the PCR product covering BMP4 open reading frame (GenBank Accession No.: NM_001202) was cloned into the pHBLV-CMVIE-ZsGreen-Puro vector to generate BMP4-overexpressed lentivirus by HanBio Technology. To establish stable BMP4-overexpression cell lines, HepG2 cells were infected with Lipofiter™ (HB-TRLF-1000, Hanbio) at 20 TU/mL MOI according to the manufacturer’s instructions. After 48 h, cells were treated with 2 μg/ml puromycin for 4 days (ant-pr-5b, InvivoGen, San Diego, CA, USA). Puromycin-resistant clones were collected and expanded for further studies. Transfection efficiency of BMP4 overexpression in HepG2 cells was confirmed by qRT-PCR and Western blot (Additional file 1: Figure S2a).

### Construction of BMP4-siRNA lentivirus and infection in HCCLM3 cells

In the experiments of BMP4 knockdown in vivo, the three candidate BMP4 siRNA and negative control were designed and constructed with a BMP4-RNA interference (RNAi) lentiviral vector (GV118-si-BMP4). The lentivirus containing si-BMP4 vector was synthesized by Genechem (Genechem Co. Ltd., Shanghai, China). The lentivirus was transfected into HCCLM3 cells with an optimal MOI of 30 TU/mL following the manufacturer’s protocol. After transfection for 72 h, cells were treated with 3 μg/ml puromycin (InvivoGen) to produce stable transfection cell lines for further experiments. Knockdown efficiency of BMP4 in HCCLM3 was acquired and confirmed by qRT-PCR and Western blot (Additional file 1: Figure S2b). The siRNA sequences with the maximum 87.3% knockdown efficiency were selected for further stable BMP4 silencing in HCCLM3 cells for animal experiments. The sequences of BMP4 siRNA are listed in Additional file 1: Table S2.

### Subcutaneous HCC tumor model in nude mice

Briefly, 5 × 10^6^ stably transfection HCCLM3 cells and HepG2 cells were suspended in 150 μL serum-free DMEM and injected subcutaneously into the left flank regions of the nude mice (4 weeks old, male, BALB/c). After cell implantation, the mice received an intraperitoneal injection of 3-MA (10 mg/kg/d) for 15 days or rapamycin (20 mg/kg/d) for 21 days. The nude mice were sacrificed after 4 weeks. The tumor masses were surgically removed and weighted. Tumor volume was calculated as follows: volume = (length × width^2^) × 0.5. All animal studies were conducted in the Animal Institute of Central South University according to the protocols approved by the Medical Experimental Animal Care Commission of the University.

### Immunohistochemistry (IHC)

The HCC tissues from the xenograft tumors were fixed in 10% formalin, dehydrated, and embedded in paraffin. After dewaxing and hydrating, antigen retrival, endogenous peroxidase activity blocking, the 4 μm-thick sections were then incubated with different primary antibodies (Ki67 1:200, LC3B 1:200, BMP4 1:400) at 4 °C overnight and followed by secondary antibodies for 30 mins at room temperature. DAB and hematoxylin staining were then performed. IHC staining intensity was performed and evaluated by the number of positive tumor cells over the total number of tumor cells.

### Statistical analysis

Statistical analysis was performed using GraphPad Prism 6.0 software. All data were presented as mean ± SD and analyzed by Student’s t-test or one-way ANOVA. Log-transformation was done with the ratio data before usual Student’s t-test or one-way ANOVA. *p* < 0.*05* was considered to be statistically significant.

## Results

### BMP4 promoted HCC proliferation in vitro

In our previous study, we have previously testified that application of BMP4 recombinant protein could enhance the proliferation of HCC cells Bel-7402 and HCCLM3 by promoting cell cycle via ID2/CDKN1B signaling [14]. But the effects of BMP4 on autophagy-regulated HCC growth remain unknown. In this study, we further investigated the effects of BMP4 on another HCC cells HepG2. Consistently, we found that the application of BMP4 recombinant protein promoted HepG2 cells growth while Noggin could effectively inhibit that (Additional file 1: Figure S3a-c), indicating the pro-growth effect of BMP4 in HCC.

### BMP4 induced autophagy in HCC cell lines

**Fig. 1:**
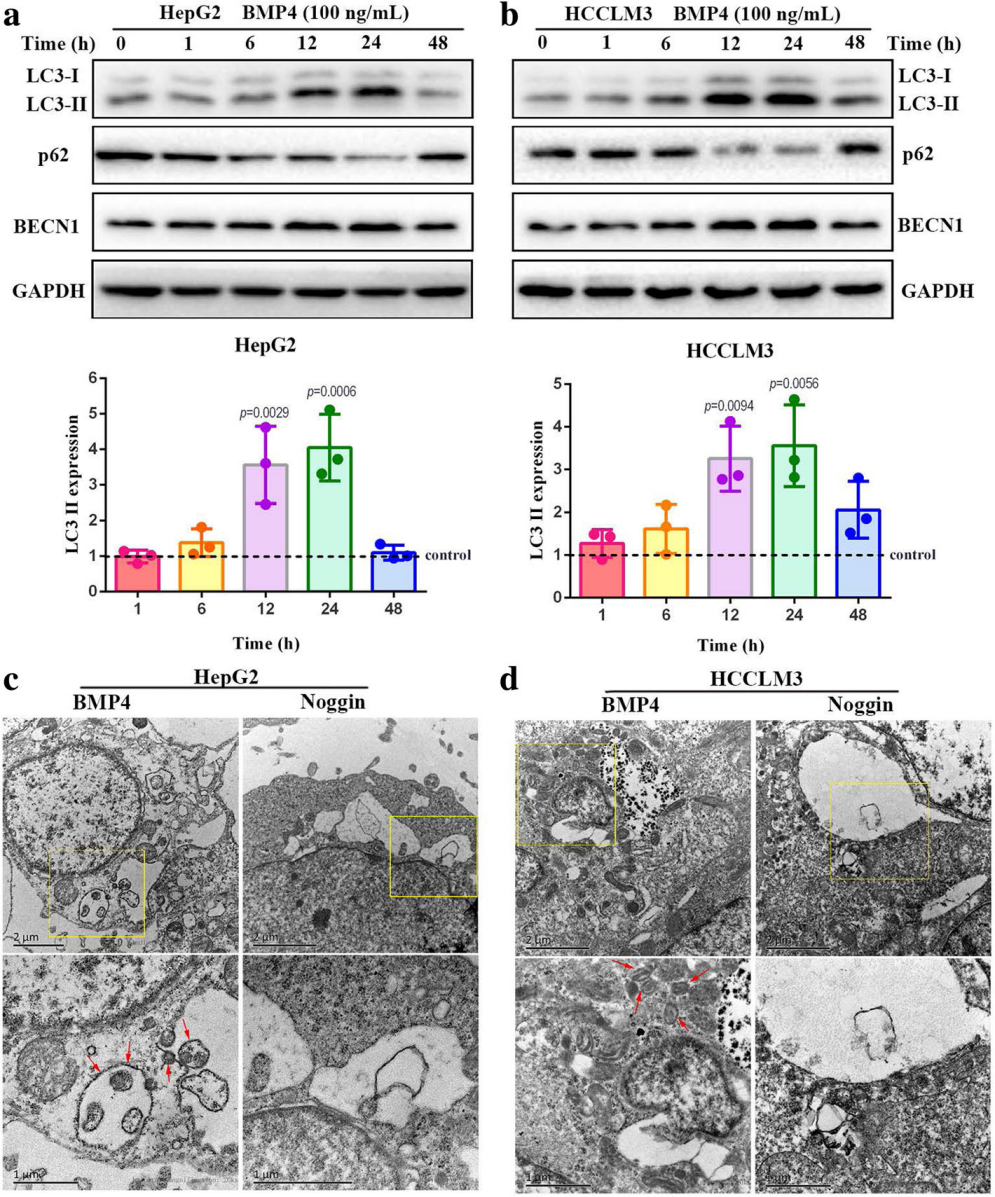
BMP4 induced autophagy in HCC cell lines: **a** & **b** HepG2 and HCCLM3 cell lines were treated with 100 ng/mL BMP4 recombinant protein for different time points (0 h, 1 h, 6 h, 12 h, 24 h and 48 h) to evaluate the effects on autophagy. Western blot was applied to detect the expression levels of LC3-II, p62 and BECN1. The expression levels of LC3-II were quantified by Image lab software by densitometric analysis and were normalized to the control groups. GAPDH was used as the internal control. *n* = 3, one-way ANOVA with post-hoc Tukey’s test. **c** & **d** Representative images of intracellular double-membrane vesicles (red arrows), the ultrastructural feature of autophagy, detected by TEM in HCC cells. HepG2 and HCCLM3 cells were treated with 100 ng/mL BMP4 or 200 ng/mL Noggin for 24 h

The effect of BMP4 on autophagy in HepG2 and HCCLM3 cells was investigated to provide insight into the molecular mechanisms responsible for BMP4-promoted HCC proliferation. We first treated HCC cells with 100 ng/mL BMP4 for varying lengths of time (1 h, 6 h, 12 h, 24 h and 48 h) and then detected autophagy marker proteins by Western blot. The increased conversion of LC3-I to LC3-II, a hallmark of autophagy, as well as an increase of Beclin-1 expression, began after 12 h and reached the most obvious effects at 24 h after the application of BMP4 recombinant protein (Fig. 1a and b, *p* < 0.01, respectively). Moreover, the Western blot indicated a gradual reduction of SQSTM1/p62 protein level in BMP4-treated HCC cells (Fig. 1a and b). The 24 h application of BMP4 recombinant protein was selected for further experiments. To further observe the autophagy activation intuitively, transmission electron microscopy (TEM) was performed. Cells after the application of BMP4 recombinant protein demonstrated greater numbers of double-membrane structures resembling autophagosomes than Noggin-treated HCC cells (Fig. 1c and d), further confirming BMP4 induced autophagy in HCC cells.

### BMP4 activated autophagic flux in HCC cell lines

**Fig. 2:**
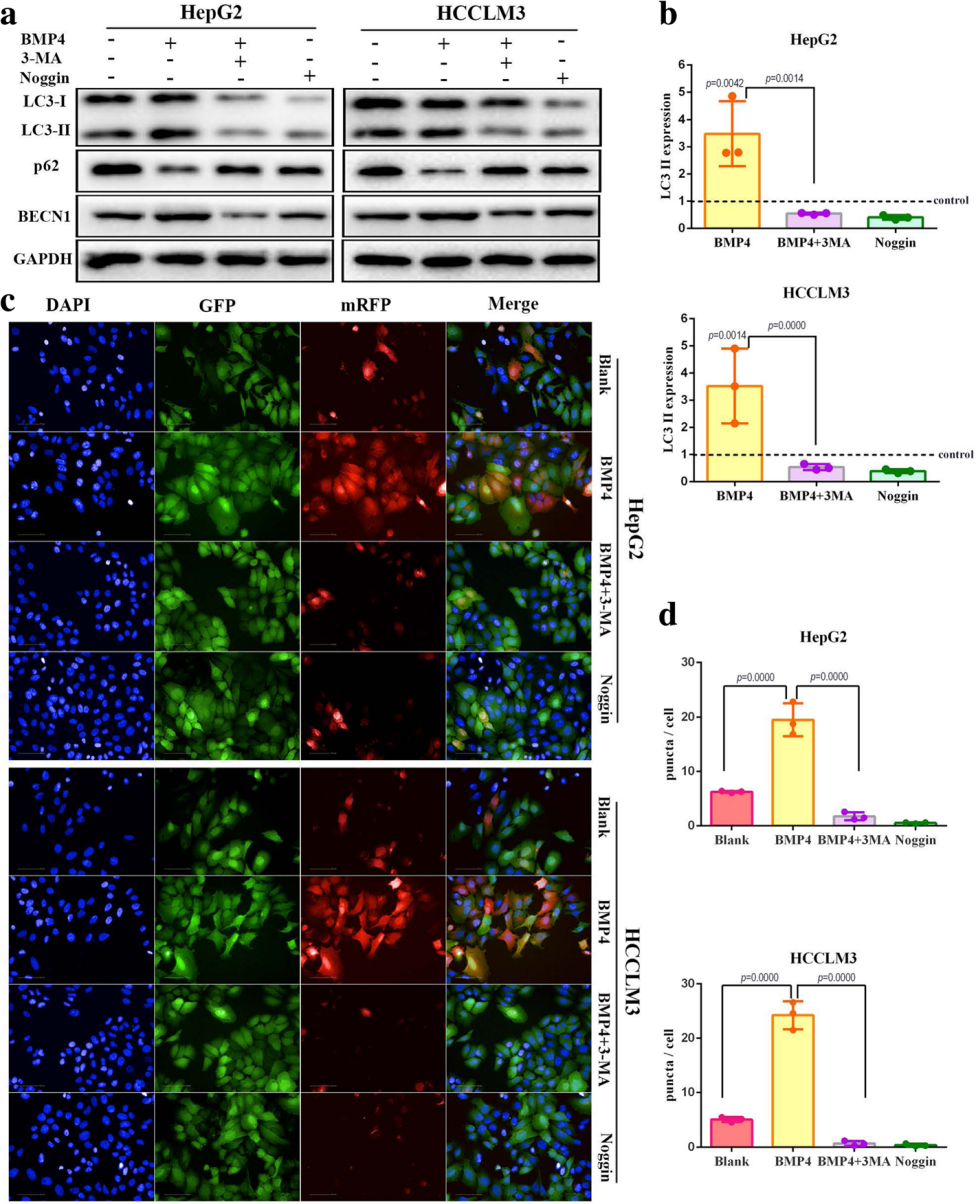
BMP4 activates autophagic flux in HCC cell lines: **a** HCC cell lines were pre-treated with autophagy inhibitor 3-MA for 1 h before BMP4 administration to prevent autophagy activated by BMP4. Western blot was applied to detect the expression levels of LC3-II, p62 and BECN1 in both HepG2 and HCCLM3 cells. **b** GAPDH was used as the internal control. Quantification analysis of LC3-II / GAPDH in Fig. 3 (**a**) was normalized to the control (blank) groups. *n* = 3, one-way ANOVA with post-hoc Tukey’s test. **c** Representative fluorescent images of mRFP-GFP-LC3 transfection. mRFP-GFP-LC3-HepG2 / HCCLM3 cells were exposed to BMP4, BMP4 with 3-MA and Noggin for 24 h. The fluorescent images were obtained from the Operetta automated microscope (Original magnification: × 200). The yellow puncta indicated autophagosomes and red puncta represent autolysosomes. **d** Quantification autophagic flux was calculated by red puncta in the merged images with Perkin-Elmer Columbus 2.3 software. *n* = 3, one-way ANOVA with post-hoc Tukey’s test

**Fig. 3:**
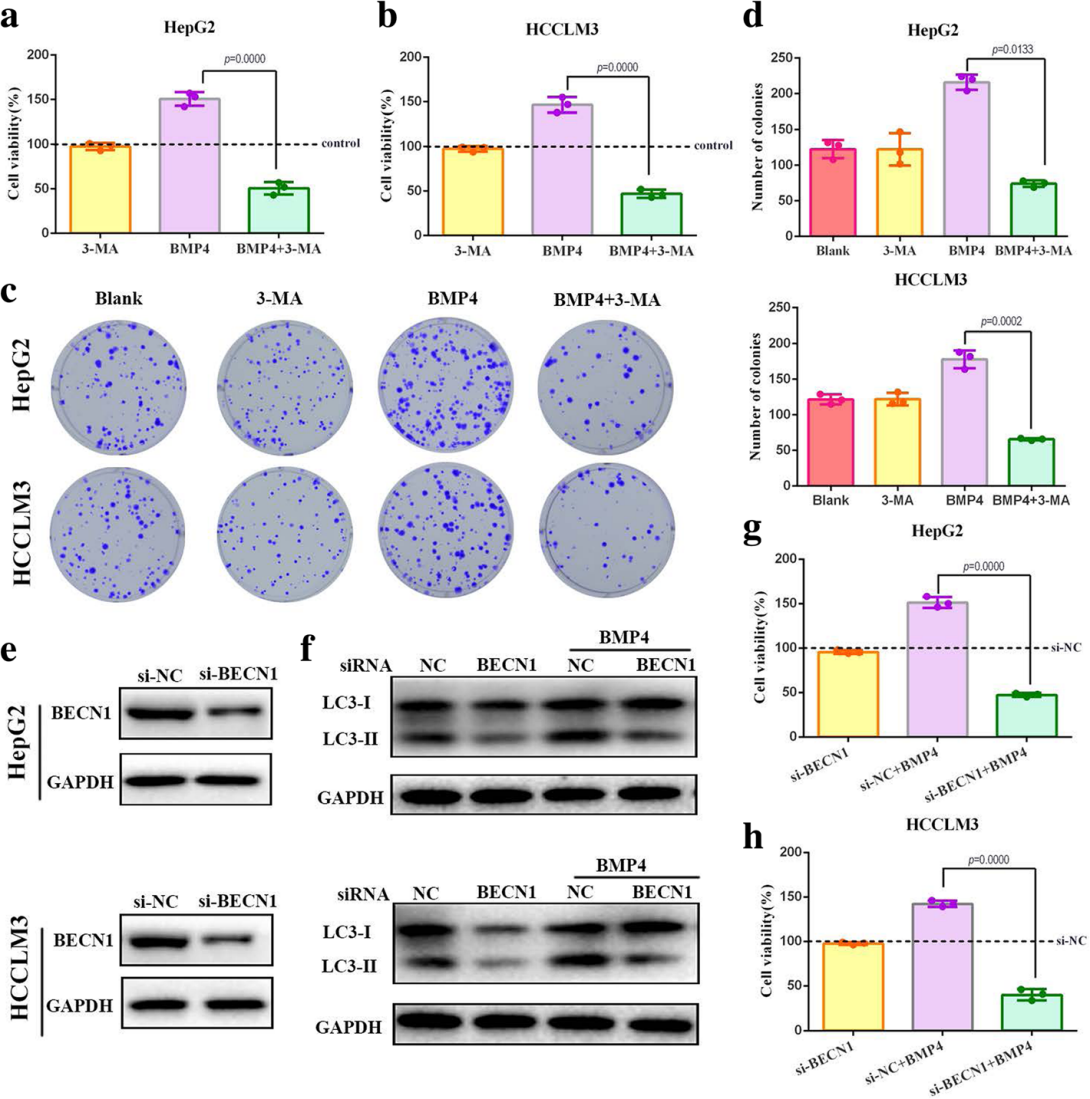
BMP4 activated-autophagy promoted HCC cells proliferation in vitro: **a** & **b** HepG2 and HCCLM3 were treated with autophagy inhibitor 3-MA, BMP4 + 3-MA and BMP4 alone. CCK-8 assays were applied to detect cell viability in HCC cells with different treatment. *n* = 3, one-way ANOVA with post-hoc Tukey’s test. **c** & **d** Effects of autophagy inhibitor 3-MA on BMP4 promoted-colony formation ability in HCC cells. Combination of 3-MA and BMP4 significantly decreased the number of colonies as compared to the BMP4-treated groups. *n* = 3, one-way ANOVA with post-hoc Tukey’s test. **e** Transfection efficiency of siRNA targeting BECN1 in HCC cells was confirmed by Western blot. **f** The expression level of LC3-II was detected by Western blot. Knockdown of BECN1 decreased the expression of LC3-II as compared with the control groups and prevented autophagy activated by BMP4. **g** & **h** Knockdown of BECN1 significantly attenuated cell viability promoted by BMP4 both in HepG2 and HCCLM3 cells. Cell viability was determined by CCK-8. *n* = 3, one-way ANOVA with post-hoc Tukey’s test

To further elucidate the effects of BMP4 on the autophagic process (autophagosome formation, fusion with lysosome and degradation in lysosome), autophagy flux was detected using a LC3-II and SQSTM1/p62 turnover assay. Application of BMP4 recombinant protein significantly promoted the expression level of LC3-II and BECN1 and decreased the expression of SQSTM1/p62 (Fig. 2a and b, *p* < 0.01, respectively). Treatment with Noggin, the antagonist of BMP4, demonstrated an opposite effect on HCC cells autophagy (Fig. 2a and b). Pre-treatment with 3-MA blocked BMP4-induced autophagy by downregulating the expression level of LC3-II and BECN1 while upregulating the expression level of SQSTM1/p62 (Fig. 2a and b, *p* < 0.01, respectively). Similar results were obtained in the analyses of mRFP-GFP-LC3 puncta distribution (Fig. 2c and d). In the blank control groups, weak signals of mRFP and GFP protein, indicators of diffuse LC3 protein, were found in the cytoplasm (Fig. 2c). After application of BMP4 recombinant protein for 24 h, yellow puncta and red puncta were observed in the perinuclear region, suggesting the formation of early autophagosomes. Combination of 3-MA and BMP4 blocked the autophagic flux induced by BMP4, representing a similar effect of BMP4 antagonist Noggin (Fig. 2c). Quantification analysis found that the mRFP-GFP-LC3 puncta numbers increased remarkably in BMP4-treated HCC cells as compared with the Blank groups (Fig. 2c and d, *p* < 0.001, respectively). 3-MA attenuated BMP4-induced autophagy flux, indicated by the significant reduction in mRFP-GFP- LC3 puncta numbers in BMP4 + 3-MA groups, compared to BMP4 groups (Fig. 3d). In addition, Noggin treatment significantly decreased the numbers of mRFP-GFP- LC3 puncta compared to the blank control groups (Fig. 2d).

### BMP4 activated-autophagy promoted HCC cells proliferation in vitro.

**Fig. 4:**
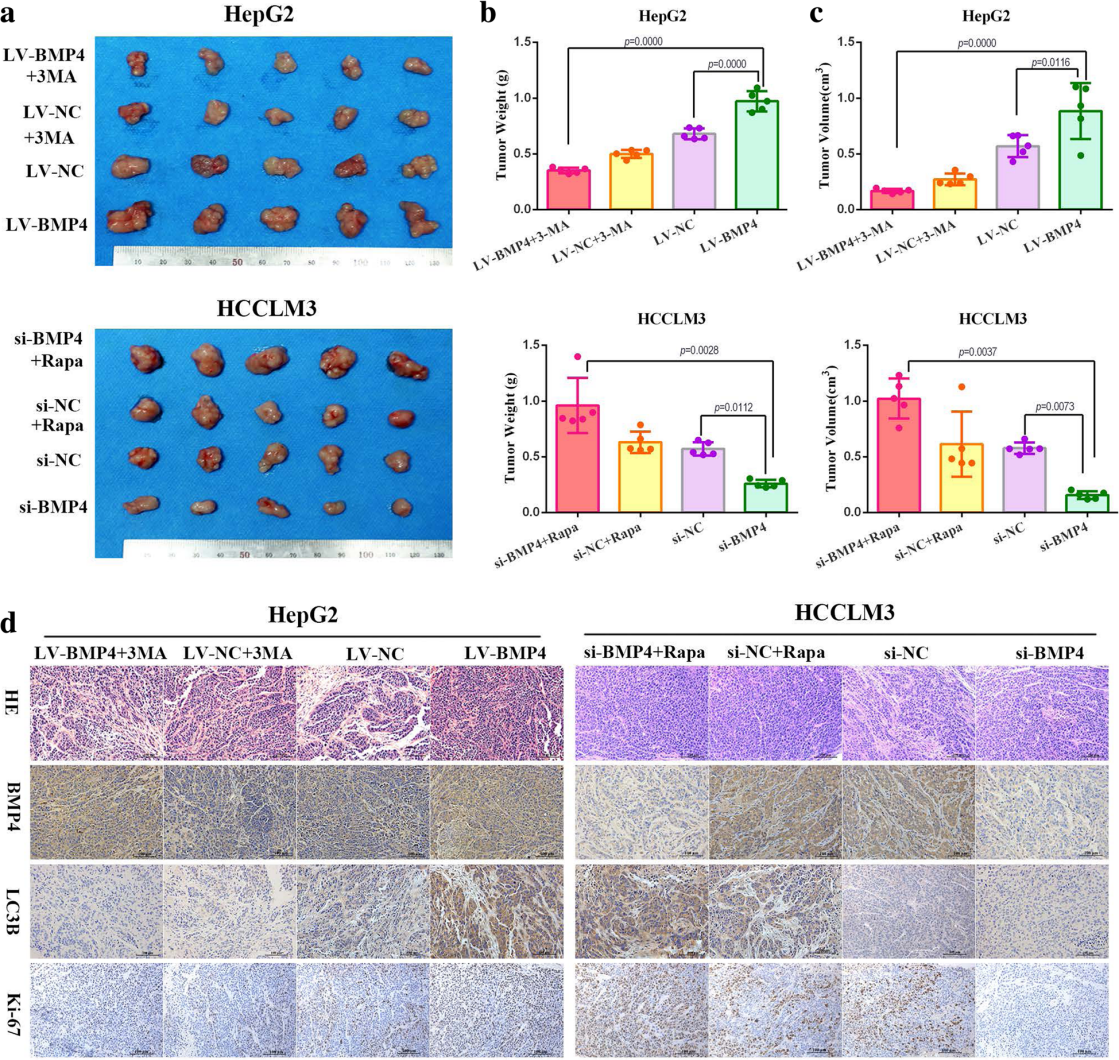
BMP4 accelerated HCC cells growth by autophagy induction in vivo: **a** upper: Tumor formation of LV-BMP4 and LV-NC treated with 3-MA in HepG2 cells; lower: Tumor formation of si-BMP4 and si-NC treated with Rapa in HCCLM3 cells. **b** The average tumor weight of BMP4 overexpression in HepG2 cells was significantly more than the control group, while knockdown of BMP4 in HCCLM3 cells decreased the tumor weight. Autophagy inhibition by 3-MA attenuated the growth-promotion effect of BMP4 overexpression while autophagy induction restored the growth-suppression effect of BMP4 knockdown. *n* = 5, one-way ANOVA with post-hoc Tukey’s test. **c** The average tumor volume of subcutaneous implantation models of ectopic BMP4 expression and autophagy manipulation in HCC cells. *n* = 5, one-way ANOVA with post-hoc Tukey’s test. **d** HE staining and immunohistochemistry staining for the expression of BMP4, LC3B and proliferation-related protein Ki-67 in subcutaneous tumor (Original magnification: × 200)

Since the effect of autophagy on cell survival is variable (a double-edged sword: protection or death induction), whether BMP4-activated autophagy was responsible for HCC cells proliferation needed to be elucidated. 3-MA was used to test whether the cell growth and colony growth promotion function of BMP4 could be abolished by blocking autophagy. HepG2 and HCCLM3 cells were treated with BMP4 recombinant protein with or without the administration of 3-MA for 24 h, and we found that 3-MA significantly attenuated the cell viability promoted by BMP4 (Fig. 3a and b). Colony formation assays further confirmed that blocking autophagy eliminated BMP4-promoted HCC growth. Co-treatment of BMP4 recombinant protein and 3-MA resulted in significantly fewer and smaller colonics than that in BMP4-treated groups (Fig. 3c and d). BECN1 is well known to regulate autophagy processes. Our previous experiments found that application of BMP4 recombinant protein upregulated the expression level of BECN1 (Fig. 1a and b). We further used siRNA targeting BECN1 to block autophagy. Knockdown of BECN1, confirmed by Western blot (Fig. 3e), effectively attenuated BMP4-promoted LC3-II conversion (Fig. 4f). Moreover, the si-BECN1 effectively eliminated BMP4-promoted HCC cells growth (Fig. 3g and h). Taken together, BMP4-promoted HCC progression could be regulated by autophagy inhibition.

### BMP4 induced autophagy to promote HCC proliferation in vivo

To further confirm the effects of BMP4 on HCC proliferation and autophagy in vivo, we established BMP4 stably overexpression cell line in HepG2 cells and BMP4 stably knockdown cell line in HCCLM3 cells for animal experiments. Rapa was used as an autophagy inducer. Four weeks after HepG2 cells implantation, the mice in the LV-BMP4 group harbored a larger average tumor weight and tumor volume than that in LV-NC group (*p* < 0.01, respectively; Fig. 4a, b and c). As compared with the LV-BMP4 group, 3-MA treatment significantly reduced the tumor weight and tumor volume (*p* < 0.001, respectively; Fig. 4a, b and c). Similar results were acquired in BMP4 knockdown HCCLM3 cells. As compared with the si-NC group, BMP4 knockdown resulted in a significant decrease in tumor weight and a remarkably smaller tumor volume (*p <* 0.01, respectively; Fig. 4a, b and c). Moreover, receiving Rapa treatment facilitated HCCLM3 cells growth and the difference of average tumor weight and average tumor volume in si-BMP4 + Rapa group was statistically significant higher than si-BMP4 group (*p <* 0.001, respectively; Fig. 4a, b and c).

**Fig. 5:**
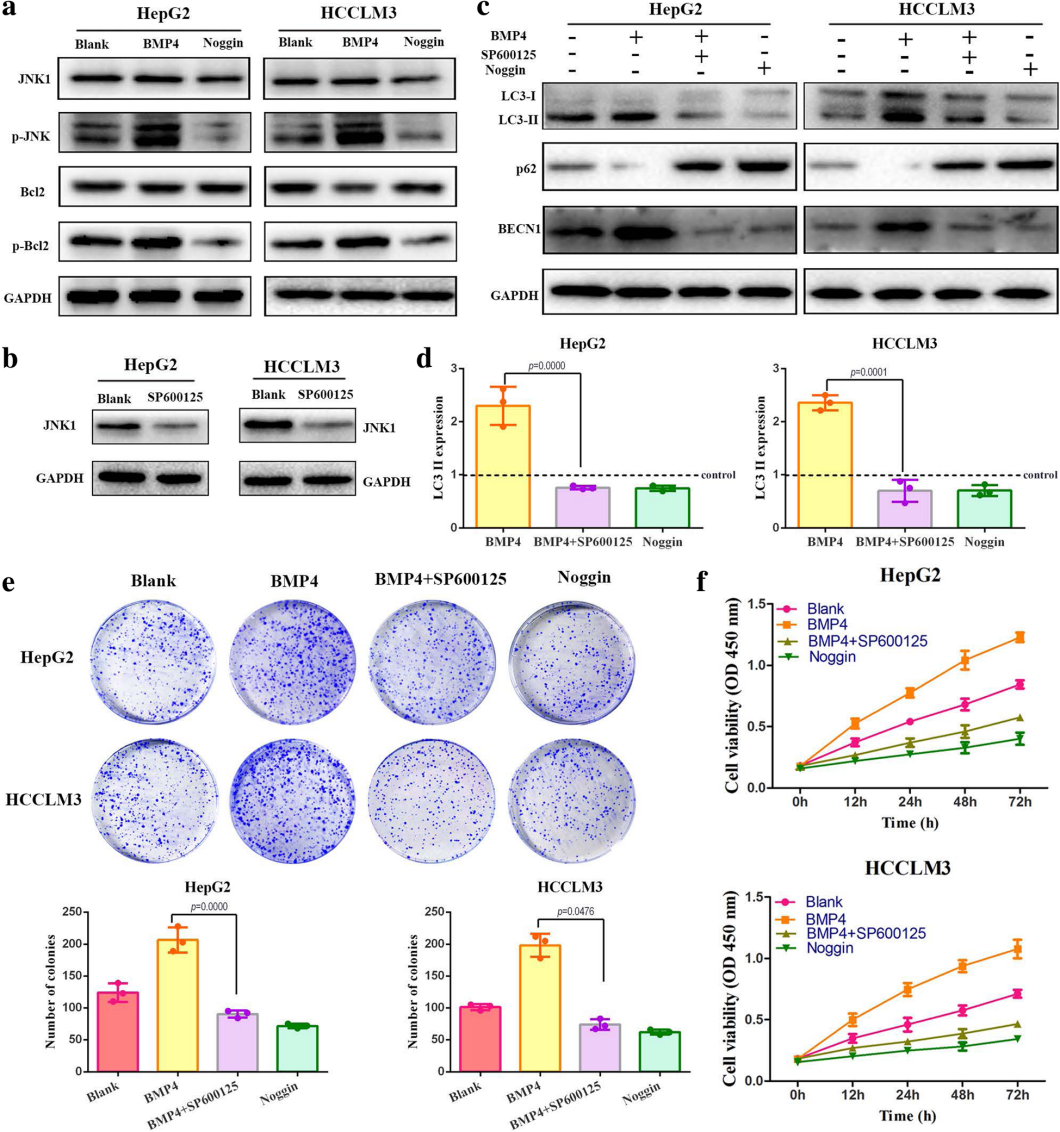
Signaling pathways involved in BMP4-activated autophagy to promote HCC proliferation: **a** Western blot was applied to detect the expression of JNK1, p-JNK, Bcl-2 and p-Bcl-2 in HepG2 and HCCLM3 cells. HCC cells were appointed to the indicated treatment respectively. **b** The effect of JNK inhibitor SP600125 was determined by Western blot in HepG2 and HCCLM3 cells. **c** Western blot was performed to detect the expression of LC3-II, p62 and BECN1 in HCC cells. JNK pathway inhibition significantly attenuated the BMP4-activated autophagy in HepG2 and HCCLM3 cells. **d** Quantification of LC3-II expression level by densitometric analysis and was normalized to the control (blank) groups. GAPDH was used as the internal control. *n* = 3, one-way ANOVA with post-hoc Tukey’s test. **e** Effects of JNK inhibitor SP600125 on long term colony formation promoted by BMP4 in HCC cells. JNK pathway inhibition significantly attenuated the promotion effects on the number of colonies both in HepG2 and HCCLM3. *n* = 3, one-way ANOVA with post-hoc Tukey’s test. **f** Effects of JNK inhibitor SP600125 on BMP4-promoted HCC cells growth. JNK pathway inhibition significantly attenuated the promotion effects on the cell viability both in HepG2 and HCCLM3

To identify the exact role of BMP4-induced autophagy in HCC growth in vivo, we further evaluated the expression levels of autophagy-related and proliferation-related makers in the ectopic BMP4 expression xenograft models. IHC assays applied to the subcutaneous tumors confirmed the overexpression and knockdown efficiency of BMP4 (Fig. 5d). BMP4 overexpression in HepG2 cells increased Ki-67 and LC3B expression while 3-MA administration attenuated the effects of BMP4-promoted growth and autophagy. Consistently, BMP4 knockdown in HCCLM3 cells leaded to a down-regulation of Ki-67 and LC3B expression. After Rapa treatment, Ki-67 and LC3B expression were significantly increased in HCCLM3 cells (Fig. 4d). Taken together, BMP4 promoted HCC cells growth and BMP4-induced autophagy contributed to HCC proliferation in vivo.

### JNK1-mediated Bcl-2 phosphorylation played an important role in BMP4-HCC proliferation

**Fig. 6:**
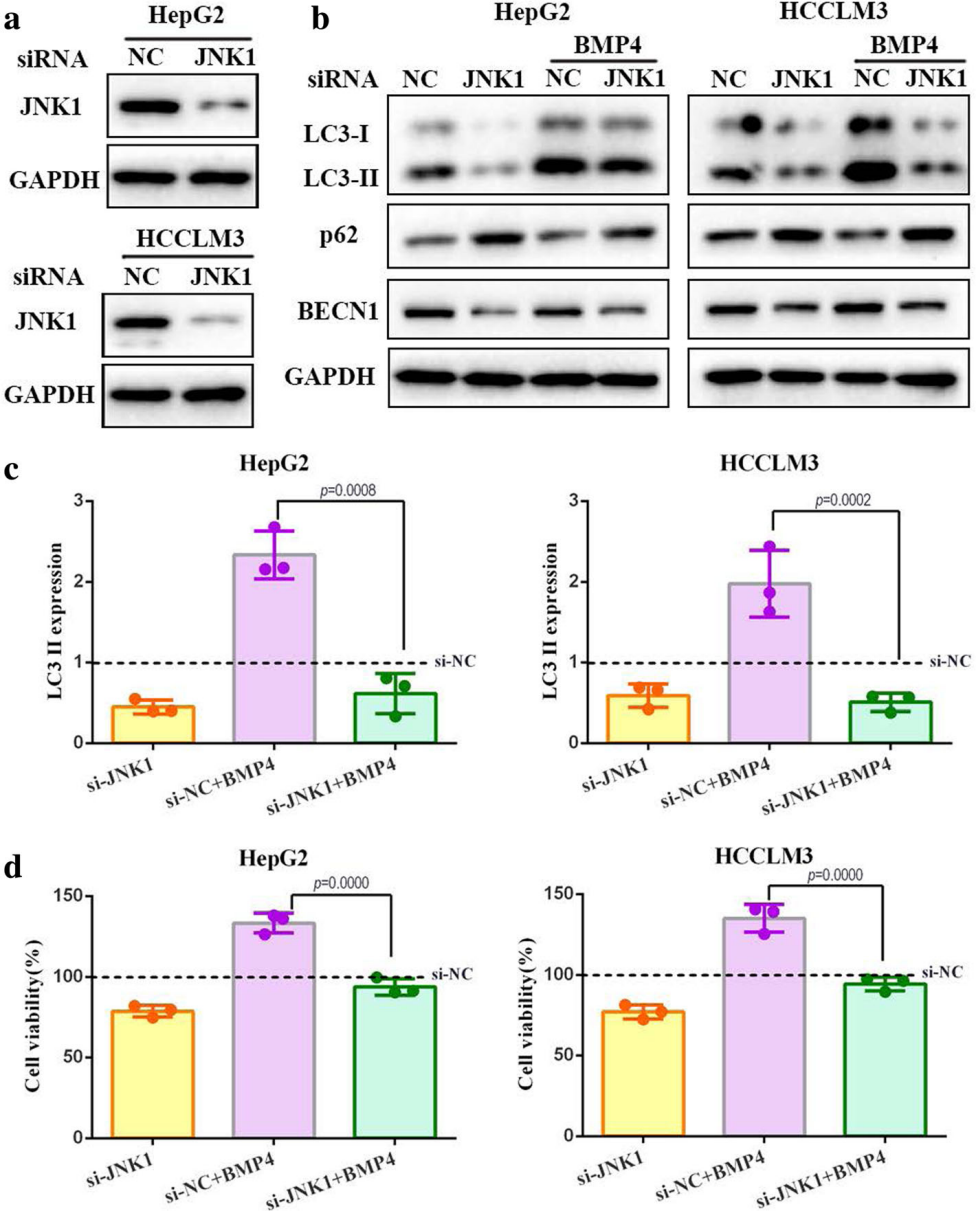
Knockdown of JNK1 attenuated BMP4-activated autophagy and HCC proliferation: **a** Transfection efficiency of siRNA targeting JNK1 in HCC cells was confirmed by Western blot. **b** The expression level of LC3-II, p62 and BECN1 were detected by Western blot. Knockdown of JNK1 decreased BMP4-promoted LC3-II conversion as compared with the BMP4-treated group. **c** The expression levels of LC3-II were quantified by Image lab software by densitometric analysis and were normalized to the control groups. GAPDH was used as the internal control. *n* = 3, one-way ANOVA with post-hoc Tukey’s test. **d** Transfected cells were treated with BMP4 for 24 h. Cell viability was determined by CCK-8. *n* = 3, one-way ANOVA with post-hoc Tukey’s test

JNK1/Bcl-2 signaling pathway has been reported to participate in cell growth and survival [18]. To determine whether this pathway is involved in BMP4-promoted HCC growth, a series of assays were performed. Cells treated with BMP4 up-regulated the expression levels of p-JNK and p-Bcl-2, while Noggin treatment exerted an opposite effect (Fig. 5a). This indicated that BMP4 activated the JNK1/Bcl-2 signaling pathway. We further investigated the relationship between JNK1/Bcl-2 signaling and BMP4-promoted HCC cells growth by using JNK inhibitor SP600125. The inhibition effects were confirmed by Western blot (Fig. 5b). The administration of JNK inhibitor with BMP4 significantly suppressed the expression of autophagic marker proteins LC3-II as compared with application of BMP4 recombinant protein alone (Fig. 5c and d, *p <* 0.001, respectively). BMP4-promoted BECN1 expression was also inhibited while BMP4-suppressed p62 expression was enhanced by JNK inhibitor (Fig. 5c). In the long term colony formation assay, the addition of JNK inhibitor remarkably decreased the number of colonics than BMP4 groups (Fig. 5e, *p <* 0.05, respectively). Moreover, the JNK inhibitor abolished BMP4-promoted HCC cells growth by decreasing cells viability (Fig. 5f). To further confirm the role of JNK1 pathway in BMP4-regulated HCC autophagy and proliferation, we utilized siRNA to knock down the expression of JNK1. Knockdown of BECN1, confirmed by Western blot (Fig. 6a), effectively attenuated BMP4-promoted LC3-II conversion (Fig. 6b and c, *p* < 0.001, respectively). Consistent with the effect of JNK inhibitor, JNK1 knockdown attenuated BMP4-promoted BECN1 expression and enhanced BMP4-inhibited p62 expression (Fig. 6b). Moreover, the si-JNK1 effectively eliminated BMP4-promoted HCC cells growth (Fig. 6d, *p* < 0.001, respectively). These data suggested that the JNK1/Bcl-2 signaling pathway is responsible for the regulation of BMP4-induced autophagy and consequential HCC growth promotion.

## Discussion

The activation of BMP signaling has been found to play an important role in various human malignancies during carcinogenesis and tumor progression. However, the effects of BMPs are complicated and depend on the type of tissue and cell context. The biological function of BMP4 on cancer cells has also aroused a great deal of controversy. Previous studies have shown that BMP4 inhibited tumorigenesis in glioblastoma, myeloma and lung cancers [19–21]. Other studies have identified BMP4 as an onco-protein with frequent overexpression in tumor tissues and positive regulation of proliferation, chemo-resistance and metastasis in several cancer cells such as the breast [22], ovarian [23], pancreatic [24], gastric [15] and colon [25] cancers. Our previous studies have found that BMP4 acted as a tumor promotion protein by facilitating HCC proliferation, invasion and chemoresistance, as well as identified its prognostic value in HCC patients [13, 14, 26]. Herein, we investigated the role of BMP4 on autophagy-regulated HCC proliferation. The data gathered in this study suggested that BMP4 promoted HCC cells growth by autophagy induction in vitro and in vivo. Autophagy flux activation was observed in the application of BMP4 recombinant protein in HCC cells while Noggin displayed an opposite effect. Autophagy inhibitor 3-MA or BECN1 knockdown attenuated HCC cells proliferation and autophagy promoted by BMP4. A mechanism exploring experiment suggested that BMP4-promoted autophagy and cell growth could be regulated by JNK1-mediated Bcl2 phosphorylation. JNK1 inhibitor and knockdown of JNK1 confirmed the above results by attenuating the promotion effects of BMP4 on HCC autophagy and cell growth.

Autophagy has been found to possess multiple functional forms: cytoprotective, cytotoxic, cytostatic, and nonprotective [27]. Basal autophagy acts as a tumor suppressor by maintaining genomic stability in normal cells. Once a tumor established, autophagy enables cancer cells survival under tumor microenvironment to promote tumor growth and development [28]. Emerging evidences suggest that autophagy is essential for cancer cells growth [29, 30], and the loss of autophagy leads to DNA damage in cancer cells by inducing reactive oxygen species (ROS) [31]. Autophagy induction contributes to cancer cells survival under stress conditions [7, 32, 33] while autophagy inhibitors suppress tumor formation in vivo [34]. It is proposed that autophagy encourages the progression of HCC through inhibiting tumor suppressors or contributing to HCC cells chemoresistance. For example, impaired-autophagy induced the expression of tumor suppressors such as p53, p21, and p27 to suppress the development of HCC [35]. In addition, some onco-proteins have been found to activate autophagy and promote tumor growth. For example, autophagy is responsible for Ras activation to maintain oxidative metabolism and tumorigenesis [36]. Consequently, blocking of autophagy might be an ideal target for therapy of established HCC. Our data demonstrated that BMP4 induced autophagy in HCC cells, with a noticeable increase of LC3-II and BECN1 expression, accompanied with a decrease of p62 expression. The autophagic flux observation confirmed the above results. Both in vitro and in vivo assays suggested that blocking of BMP4-induced autophagy suppressed HCC proliferation.

Cell death is a tightly regulated biological process where autophagy and apoptosis both play a central role. The relationship between autophagy and apoptosis is complex and regulators of apoptosis activation also function as regulators of autophagy activation. BECN1, autophagy-related gene 6, is part of a Type III PI3 kinase complex which is required for the autophagosome formation and mediates the localization of other autophagy proteins to the pre-autophagosomal membrane [37]. BECN1 was initially identified to regulate autophagy and interfered with BECN1 to prevent autophagy induction [38, 39]. In addition, Bcl-2, a major anti-apoptotic protein of Bcl family, was found to interact with BECN1 and was thought to be the crosstalk between cell autophagy and apoptosis. By binding to BECN1, Bcl-2 exerts its autophagy inhibition function, suggesting that the dissociation of Bcl-2 from BECN1 may be an important mechanism for activating autophagy [40]. The c-Jun N-terminal protein kinase (JNK) is a member of the mitogen-activated protein kinase (MAPK) superfamily and has been demonstrated to occupy a critical position in both autophagy and apoptosis processes. It was suggested that JNK1-mediated phosphorylation of Bcl-2, dissociated BECN1 from BECN1-Bcl-2 complex and resulted in autophagy activation [41]. We found that the JNK1 was activated under BMP4 treatment while Noggin blocked the activation of JNK1. Subsequently the phosphorylation of Bcl-2 was increased in the BMP4-treated groups whereas cells in the Noggin-treated groups inhibited the Bcl-2 phosphorylation, indicating the JNK1/Bcl-2 signaling might be a crucial mechanism involved in BMP4-promoted HCC cells growth. As we envisaged, when JNK1 inhibitor was added together with BMP4 treatment, the autophagy activity and cell viability enhanced by BMP4 were attenuated. Similarly, BMP4-promoted autophagy activity and cell viability were attenuated by knockdown of JNK1. Further functional experimental results confirmed our hypothesis. These results indicated that activation of JNK1/ Bcl-2 signaling pathway played an important role in the induction of autophagy by BMP4 to promote HCC cells growth. However, we did not explore the regulation of other BECN1 interactome such as BECN1/VPS34 complex. It will be important to explore more detailed mechanisms in further study.

In summary, our study suggested that BMP4 acted as a proliferation-promoted molecular and this effect was exerted by autophagy induction in HCC cells in vitro and in vivo. BMP4-activated JNK/Bcl-2 signaling pathway faciliated the proliferation of HCC cells. Further in-depth studies are needed to uncover the role of autophagy in BMP4-targeted therapy, which will benefit HCC patients through development of novel treatment regimen and provide the basis for the design of multiple clinical trials.

## Conclusions

In this study, we offer convincing evidences that BMP4 exerted oncogenic effects in HCC cells. These effects were demonstrated by its ability to induce autophagy and facilitate HCC cells growth which were dependent on BECN1 through activating JNK1/Bcl2 pathway. Together, it indicated a new mechanism by which BMP4 regulated autophagy to participate in HCC progression.

## Additional file

Additional file 1: **Figure S1.** Original blots of Western blot. Original blots of Fig. 1. (2) Original blots of Fig. 2. (3) Orinigal blots of Fig. 3. (4) Orinigal blots of Fig. 4. (5) Orinigal blots of Fig. 5. (6) Orinigal blots of Fig. 6. **Figure S2**. The effeciency of BMP4 expression manipulated by lentivirus. The effeciency of BMP4 overexpression in HepG2 cells was confirmed by qRT-PCR and Western blot. (b)The effeciency of BMP4 knockdown in HCCLM3 cells was confirmed by qRT-PCR and Western blot. **Figure S3.** BMP4 promoted HepG2 cells growth. (a) HepG2 cells were treated with various concentrations of human recombinant BMP4 (0, 25, 50, 100 and 150 ng/mL) for different lengths of time to assay the effects on HCC cells proliferation. Cell viability was determined by CCK-8 assays. (b) & (c) Effects of BMP4 or Noggin on long term colony formation in HepG2 cells. The numbers of colonies in the BMP4-treated (100 ng/mL) groups were significantly more than that in the blank groups in HepG2 cells (*p* < 0.001), while Noggin-treated (200 ng/mL) groups displayed significantly less colony numbers than the blank control groups (*p* < 0.05). *n* = 3, one-way ANOVA with post-hoc Tukey’s test. **Table S1.** Information of antibodies for western blot. **Table S2**. The sequences of siRNA targeting BMP4. (PDF 783 kb)

## Abbreviations

3-MA: 3-Methyladenine
BECN1: Beclin-1
BMP4: Bone morphogenetic protein 4
FBS: Fetal bovine serum
HCC: Hepatocellular carcinoma
HSCs: Hepatic stellate cells
JNK: c-Jun N-terminal protein kinase
LV: Lentivirus
MAPK: Mitogen-activated protein kinase
Rapa: Rapamycin
ROS: Reactive oxygen species
siRNA: Small interfering RNA
TEM: Transmission electron microscopy
TGF-β: Transforming growth factor β
