# Supplementary material for: BMP4 promotes hepatocellular carcinoma proliferation by autophagy activation through JNK1-mediated Bcl-2 phosphorylation
Source: J Exp Clin Cancer Res. 2018 Jul 16;37:156. doi: 10.1186/s13046-018-0828-x (PMC6048721; doi:10.1186/s13046-018-0828-x)
Supplement: Supplementary file 1 — Figure S1. Original blots of Western blot. Original blots of Fig. 1. (2) Original blots of Fig. 2. (3) Orinigal blots of Fig. 3. (4) Orinigal blots of Fig. 4. (5) Orinigal blots of Fig. 5. (6) Orinigal blots of Fig. 6. Figure S2. The effeciency of BMP4 expression manipulated by lentivirus. The effeciency of BMP4 overexpression in HepG2 cells was confirmed by qRT-PCR and Western blot. (b)The effeciency of BMP4 knockdown in HCCLM3 cells was confirmed by qRT-PCR and Western blot. Figure S3. BMP4 promoted HepG2 cells growth. (a) HepG2 cells were treated with various concentrations of human recombinant BMP4 (0, 25, 50, 100 and 150 ng/mL) for different lengths of time to assay the effects on HCC cells proliferation. Cell viability was determined by CCK-8 assays. (b) & (c) Effects of BMP4 or Noggin on long term colony formation in HepG2 cells. The numbers of colonies in the BMP4-treated (100 ng/mL) groups were significantly more than that in the blank groups in HepG2 cells (p < 0.001), while Noggin-treated (200 ng/mL) groups displayed significantly less colony numbers than the blank control groups (p < 0.05). n = 3, one-way ANOVA with post-hoc Tukey’s test. Table S1. Information of antibodies for western blot. Table S2. The sequences of siRNA targeting BMP4. (PDF 783 kb) [file 13046_2018_828_MOESM1_ESM.pdf]

BMP4 promotes hepatocellular carcinoma proliferation by autophagy activation through JNK1-mediated Bcl-2 phosphorylation

Author name: Ganlu Deng [1, 2], Shan Zeng [1, 3,] Yanli Qu [1, 2], Qingqing Luo [1, 2], Cao Guo [1, 3], Ling Yin [1, 2,] Ying Han [1, 2], Yiyi Li [1, 2], Changjing Cai [1, 2], Yaojie Fu[1, 2], Hong Shen [1, 2, 3, *]

Authors' affiliations:
1 Department of Oncology, 2 National Clinical Research Center for Geriatric Disorders,
3 Key Laboratory for Molecular Radiation Oncology of Hunan Province,
Xiangya Hospital, Central South University, Changsha, Hunan, China 410008

* Address reprint requests to: Dr. Hong Shen,

Institute of Medical Sciences, Xiangya Hospital,

Central South University, Changsha, Hunan, China 410008.

Tel: (86) 731-8432-7633

E-mail: hongshen2000@csu.edu.cn

Supplementary Information include:
Supplementary Figure S1-S3 and supplementary Table S1-S2.

Figure S1

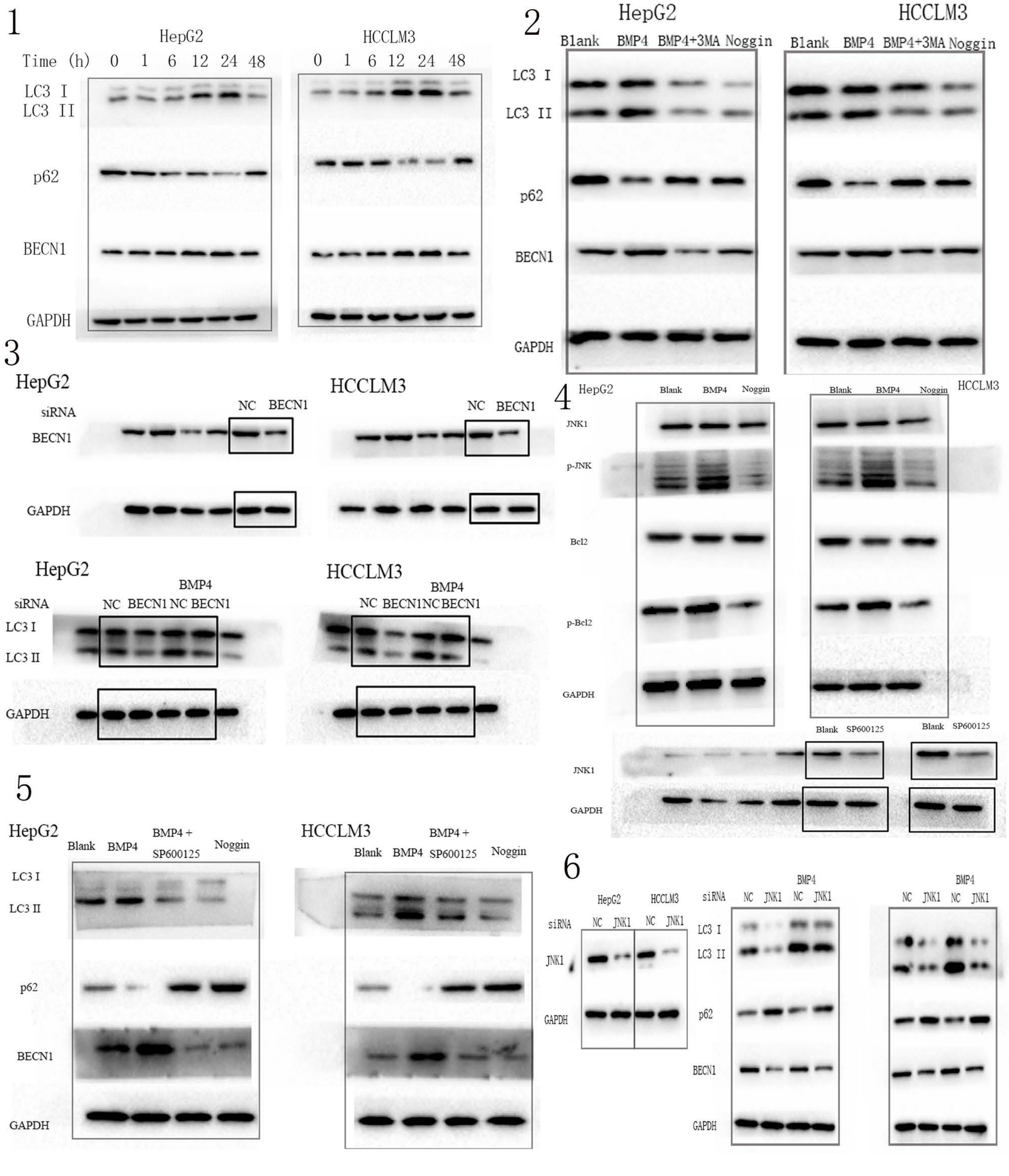

Figure S1: Original blots of Western blot. (1) Original blots of Figure 1. (2) Original blots of Figure 2. (3) Orinigal blots of Figure 3. (4) Orinigal blots of Figure 4. (5) Orinigal blots of Figure 5. (6) Orinigal blots of Figure 6.

Figure S2

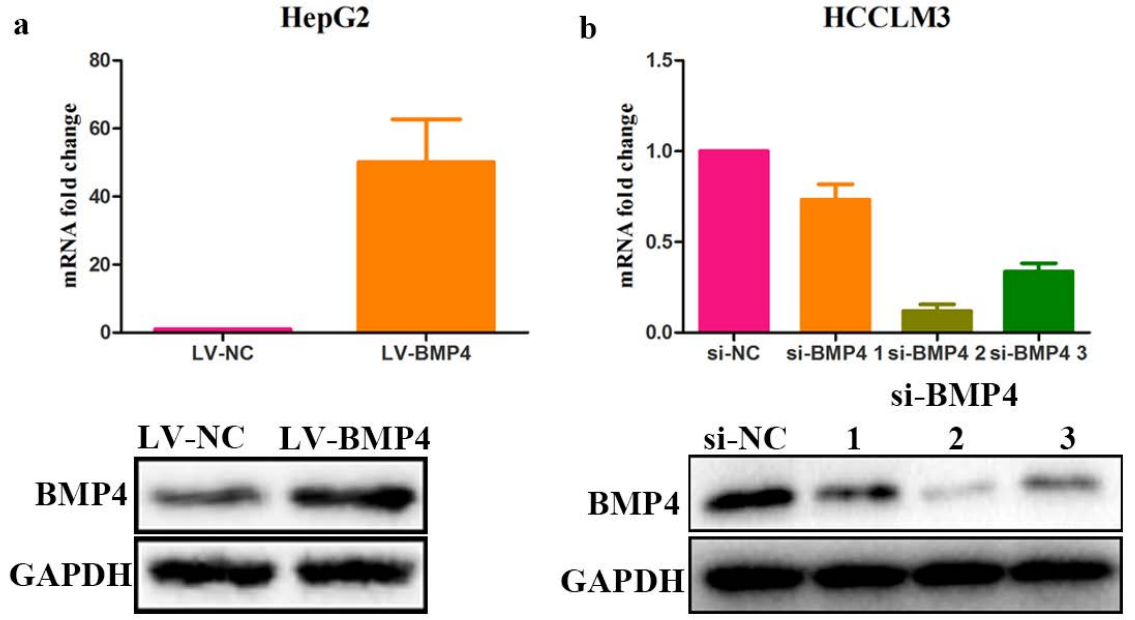

Figure S2: The effeciency of BMP4 expression manipulated by lentivirus. (a)The effeciency of BMP4 overexpression in HepG2 cells was confirmed by qRT-PCR and Western blot. (b)The effeciency of BMP4 knockdown in HCCLM3 cells was confirmed by qRT-PCR and Western blot.

Figure S3

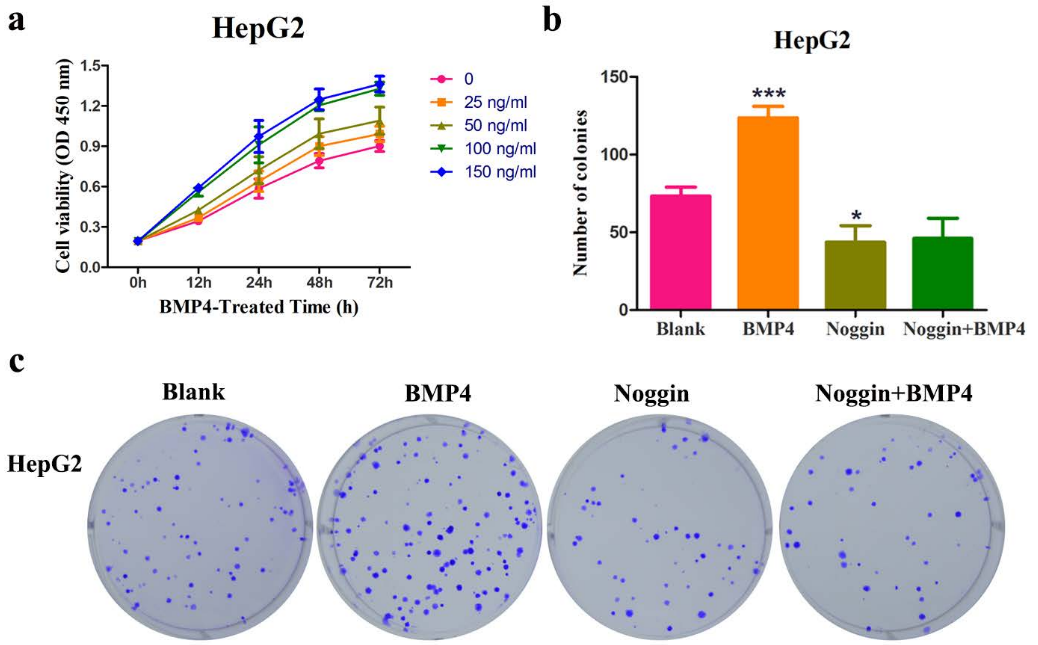

Figure S3: BMP4 promoted HepG2 cells growth: (a) HepG2 cells were treated with various concentrations of human recombinant BMP4 (0, 25, 50, 100 and 150 ng/mL) for different lengths of time to assay the effects on HCC cells proliferation. Cell viability was determined by CCK-8 assays. (b) & (c) Effects of BMP4 or Noggin on long term colony formation in HepG2 cells. The numbers of colonies in the BMP4-treated (100 ng/mL) groups were significantly more than that in the blank groups in HepG2 cells ($p < 0.001$), while Noggin-treated (200 ng/mL) groups displayed significantly less colony numbers than the blank control groups ($p < 0.05$). n=3, one-way ANOVA with post-hoc Tukey's test.

Supplementary Table S1. Information of antibodies for western blot.

Antibody	Working Dilution	Molecular Weight (KDa)	Cat. Number
BMP4	1:5000	47	ab124715
LC3	1:1000	14, 16	#4108
SQSTM1/p62	1:1000	62	ab56416
Beclin1	1:1000	60	ab207612
JNK1	1:1000	48	ab213521
phospho-JNK	1:1000	46, 54	#4668
Bcl-2	1:1000	26	#4223
phospho-Bcl2	1:1000	28	#2827
GAPDH	1:10000	36	ab181602
Goat anti-Mouse IgG	1:5000	—	ab6789
Goat anti-Rabbit IgG	1:5000	—	ab6721

Supplementary Table S2. The sequences of siRNA targeting BMP4.

No.	sequences	titer
BMP4-RNAi1 - 5’	taaGCAGCCAAACTATGGGCTA<br>ctcgagTAGCCCATAGTTTGGCTGCttttttttc	5E+8
BMP4-RNAi1 - 3’	tcgagaaaaaaaaGCAGCCAAACTATGGGCTA<br>ctcgagTAGCCCATAGTTTGGCTGCtta	5E+8
BMP4-RNAi2 - 5’	tgaCCCTGGTCAATTCTGTCAA<br>ctcgagTTGACAGAATTGACCAGGGtcttttttc	4E+8
BMP4-RNAi2 - 3’	tcgagaaaaaagaCCCTGGTCAATTCTGTCAA<br>ctcgagTTGACAGAATTGACCAGGGtca	4E+8
BMP4-RNAi3 - 5’	tcaCCTCAACTCAACCAACCAT<br>ctcgagATGGTTGGTTGAGTTGAGGtgttttttc	5E+8
BMP4-RNAi3 - 3’	tcgagaaaaaacaCCTCAACTCAACCAACCAT<br>ctcgagATGGTTGGTTGAGTTGAGGtga	5E+8
BMP4-RNAi4 - 5’	tcaGATCCACAGCACTGGTCTT<br>ctcgagAAGACCAGTGCTGTGGATCtgttttttc	3E+8
BMP4-RNAi4 - 3’	tcgagaaaaaacaGATCCACAGCACTGGTCTT<br>ctcgagAAGACCAGTGCTGTGGATCtga	3E+8
